# Contrast-enhanced Micro-CT 3D visualization of cell distribution in hydrated human cornea

**DOI:** 10.1016/j.heliyon.2024.e25828

**Published:** 2024-02-03

**Authors:** Gerard Boix-Lemonche, Torben Hildebrand, Håvard Jostein Haugen, Goran Petrovski, Liebert Parreiras Nogueira

**Affiliations:** a Center for Eye Research and Innovative Diagnostics, Department of Ophthalmology, Institute for Clinical Medicine, Faculty of Medicine, University of Oslo, Norway; b Department of Biomaterials, University of Oslo, Norway; c Oral Research Laboratory, Institute of Clinical Dentistry, Faculty of Dentistry, University of Oslo, Oslo, Norway; d Department of Ophthalmology, and Norwegian Center for Stem Cell Research, Oslo University Hospital, Oslo, Norway; e Department of Ophthalmology, University of Split School of Medicine and University Hospital Centre, Split, Croatia; f UKLO Network, University St. Kliment Ohridski – Bitola, Bitola, Macedonia

**Keywords:** Cornea, Contrast-enhanced micro-CT, Swelling, Collagen fibrils, Virtual histology

## Abstract

**Background:**

The cornea, a vital component of the human eye, plays a crucial role in maintaining visual clarity. Understanding its ultrastructural organization and cell distribution is fundamental for elucidating corneal physiology and pathology. This study comprehensively examines the microarchitecture of the hydrated human cornea using contrast-enhanced micro-computed tomography (micro-CT).

**Method:**

Fresh human corneal specimens were carefully prepared and hydrated to mimic their *in vivo* state. Contrast enhancement with Lugol's iodine-enabled high-resolution Micro-CT imaging. The cells' three-dimensional (3D) distribution within the cornea was reconstructed and analyzed.

**Results:**

The micro-CT imaging revealed exquisite details of the corneal ultrastructure, including the spatial arrangement of cells throughout its depth. This novel approach allowed for the visualization of cells' density and distribution in different corneal layers. Notably, our findings highlighted variations in cell distribution between non-hydrated and hydrated corneas.

**Conclusions:**

This study demonstrates the potential of contrast-enhanced micro-CT as a valuable tool for non-destructive, 3D visualization and quantitative analysis of cell distribution in hydrated human corneas. These insights contribute to a better understanding of corneal physiology and may have implications for research in corneal diseases and tissue engineering.

## Key Highlights

• **3D Imaging of Corneal Structure:** Comprehensive 3D visualization of the corneal structure, enabling in-depth analysis.
• **Contrast-Enhanced Micro-CT with Lugol's Iodine:** Contrast agent for micro-CT, enhancing imaging precision.
• **Hydration-Induced Structural Changes:** Alterations in the corneal epithelium, a critical finding for corneal research.
• **Virtual Histology for Digital Sectioning:** Virtual histology to facilitate cross-sectional views of thin layers.
• **Developed method for H&E-like appearance:** Enhanced visualization of micro-CT scans through color mapping.

## Graphical abstract

**Figure:**
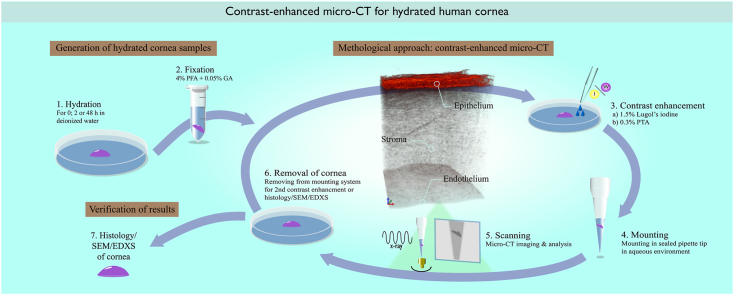

## 1 Introduction

The human cornea is a transparent avascular tissue, a multi-layered component of the ocular surface. It has a crucial role in the transmission and the focus of light onto the lens, where it is transmitted towards the retina [[1], [2], [3]]. It is an essential structure for vision, and damage to the cornea can lead to vision loss. The transparency of the cornea is regulated by the uniform organization of the collagen fibrils (CFs) [4,5], which are maintained by the proteoglycans (PGs) [6]. Additionally, the endothelium role is crucial in maintaining the proper balance of fluid entering and leaving the stroma [7]. Adequate hydration of the CFs contributes to its uniform distribution, which is necessary for the cornea's transparency. Also, the PGs can regulate this hydration, which helps ensure that the CFs are distributed uniformly [5,8].

The cornea's stroma is composed of collagen fibril lamellae, which are interconnected to each other in the anterior stroma. The anterior stroma is the most significant and stable human cornea region, providing biomechanical strength and UV-B protection [5]. Some researchers predicted that the anterior stroma in humans would scatter almost twice as much light per unit depth as the posterior stroma, possibly due to the organization in the anterior-posterior spatial ordering fibrils [9]. Since the human cornea's anterior stroma is structured specifically with lamellae and CFs, excessive moisture has no negative effects [7]. Several researchers have investigated the effect of swelling on the structure of the corneal stroma in various mammal species [5,[10], [11], [12], [13], [14]]. It has been hypothesized that water can not only fill the space between CFs but also enter the CFs during the swelling of the corneal stroma [10]. The endothelial pump activity contributes to the swelling's mechanism. The cornea becomes opaque when the endothelial pump cannot operate normally due to excessive moisture [15,16]. However, some authors suggest that under excessive hydration, degraded CFs congregate and produce massive fibril complexes [17]. Other researchers who studied the effect of PBS and deionized water on human corneal swelling observed that. In contrast, the anterior stroma of the human cornea did not swell, the middle and posterior stroma of the cornea did, and the interfibrillar spacing expanded in the hydrated corneas while the CF's diameter remained unaffected [7,18,19]. Currently, diagnostic tests for corneal swelling are completed in an outpatient setting and may include an eye exam and the use of magnifying tools such as a slit lamp or ophthalmoscope [20]. Treatment options for corneal swelling depend on the underlying cause and may include medications, surgery, or the use of contact lenses [20].

Nano-CT (also known as micro-CT or microcomputed tomography) is a non-destructive imaging technique that uses X-rays to produce high-resolution, three-dimensional images of tissues and other small specimens [21]. In addition, nano-CT can visualize the internal microstructure of tissues in great detail, including the arrangement and size of cells and other structures.

Although nano-CT has been used to study the ultrastructure of tissues and organs, such as the lungs, liver, and bones [[22], [23], [24]], it has never been used to examine human corneas. Nano-CT can be used to investigate the ultrastructure of small biological specimens, such as cells and tissues that have been fixed and prepared for imaging.

One advantage of this visualization technique is that it can produce images with a high spatial resolution, typically in the range of a few hundred nanometers to a few micrometers [25,26]. Using different imaging techniques allows visualization of small parts and structures within corneal tissue that may not be visible otherwise.

Nano-CT can therefore, be used in research and clinical applications, including drug development, tissue engineering, and the study of corneal diseases. Understanding corneal biomechanics and 3D reconstruction of corneal- and other tissue-ultrastructural features in all cross-sections and degrees of angle can support disease diagnostics and prognostics. The front and the back part of the corneal stroma have different scattering properties of light due to spatial differences in the ordering of the fibrils [9].

The present study investigated the ultrastructural changes of the cornea and its CFs and cells of the corneal epithelium, stroma, and endothelium under swelling conditions. The CF density, interfibrillar spacing, and the stroma's cellular distribution were investigated for the first time using nano-CT and 3D ultrastructural visualization technology in the human cornea.

## 2 Material and methods

### 2.1 Sample preparation

Human tissue procurement as leftover donor tissue for research purposes from the local Cornea Bank following the tenets of the Declaration of Helsinki and approval by the Regional Committees for Medical and Health Research Ethics, Norway (REK: 2017/418). Four healthy human corneas were used for this study and divided accordingly. One part of the corneas was hydrated individually in deionized water to induce swelling for 2 h and 48 h, while the remaining healthy/untreated corneas were used as a control. The corneas were fixed in a mixture of 4% paraformaldehyde+0.05% glutaraldehyde immediately after removal from the deionized water.

### 2.2 Micro-computed tomography (Micro-CT)

The corneal tissue was cut into samples with a dimension of about 2 × 2 mm and the given thickness using a scalpel under an aqueous environment. The specimens were incubated in 1.5% Lugol's iodine (iodine/potassium 1:2) and for the second scan, additionally in 0.3% phosphotungstic acid (PTA) in water for each 24 h, rinsed in water and then placed inside a sealed 200 μL pipette tip in water (Mettler-Toledo Rainin, Oakland, CA, USA). All specimens contrast-enhanced with Lugol's iodine were scanned by a nano-CT (SkyScan 2211 Multiscale X-ray Nano-CT System, Bruker micro-CT, Kontich, Belgium) with a 20–190 kV tungsten X-ray source and a dual detection system: an 11- megapixel cooled 4032 × 2670-pixel CCD-camera and a 3-megapixel 1920 × 1536 pixel CMOS flat panel. The specimens were scanned at 40 kV, 300 μA and 800 ms over 180° with a rotation step of 0.13°, leading to a final voxel size of 0.8 μm. Specimen stained with PTA were scanned by a micro-CT (Skyscan 1172, Bruker micro-CT, Kontich, Belgium) at 50 kV, 160 μA, and 780 ms exposure time over 360° with a rotation step of 0.31°, leading to a final voxel size of 1.62 μm. The scan duration for samples was about 1 h. Nano and micro-CT projections were reconstructed using the system-provided software NRecon (version 1.7.4.6) with smoothing kernel 2, ring artifact correction 12, and beam hardening correction of 50% for nano-CT scans and ring artifact correction 10, and beam hardening correction of 40% for micro-CT scans. The 3D image sets were visualized and analyzed with Dragonfly (Object Research Systems (ORS), Montréal, Canada, version 2022.1).

### 2.3 Synchrotron X‐ray computed micro‐tomography

The corneal tissue was cut into samples with a dimension of about 2 × 2 mm and the given thickness using a scalpel under an aqueous environment. The specimens were incubated in 1.5% Lugol's iodine (iodine/potassium 1:2) or remained without contrast enhancement. Subsequently, the specimen underwent stepwise dehydration in ethanol, followed by paraffin wax embedding after clearing with xylene and mounting in a 200 μL pipette tip (Mettler-Toledo Rainin, Oakland, CA, USA). Samples were scanned at the SYRMEP beamline in the synchrotron laboratory ELETTRA (Trieste, Italy) using a sample-to-detector distance of 100 mm and a 0.5 mm silicon filter. The projections were obtained with 16.7 keV and 308 mA and an exposure time of 50 ms over 180°. The pixel size was set to 0.9 μm. Acquisition times were approximately 4 min for each scan. Reconstructions were performed using STP (version 1.6.2), a custom reconstruction program of SYRMEP. Analysis was performed in Dragonfly (Object Research Systems (ORS), Montréal, Canada, version 2022.1).

### 2.4 Structural analysis of corneal tissue

Based on the obtained projections and contrasts, the thickness of the corneal tissues (epithelium, stroma, and endothelium) was determined by the specimen scanned with prior contrast enhancement with 1.5% Lugol's iodine. Epi- and endothelium were segmented by selecting an adequate range of grey values, followed by a cleaning up to remove unintended segmentations, including the corresponding other tissue and fill of inner areas in the segmented layer. The thickness was then calculated by creating a “volume thickness map” of the segmentation. Stromal thickness was defined as the spatial distance between epi- and endothelium and determined by creating a “distance map” of the endothelium and calculating the distance to the epithelium.

Determination of the porosities of the stroma was performed on scans with subsequent contrast enhancement with PTA. A cylinder with a diameter of 250 μm and the greatest possible height, not enclosing endo- or epithelium, was selected for sampling, followed by segmenting the intermediate space and stromal tissue by an adequate range of grey values. Porosity is then defined as the volume ratio of intermediate space and stromal tissue.

### 2.5 Evaluation of the contrast enhancement

Contrast-to-Noise ratio (CNR) was calculated with Equation (1) for endothelium, epithelium, and stromal tissue for iodine- and PTA contrast-enhanced projections. For signal values ($S_{A}$ and corresponding noise $\sigma_{A}$), a segmentation of the endothelium and the epithelium was used. Stromal grey values were determined by sampling in the ROI using a cylinder with a diameter of 250 μm and the greatest possible height, not enclosing endo- or epithelium. A sphere with a diameter of 250 μm close to the sample was used as a background reference ($S_{B}$ and corresponding noise $\sigma_{B}$).

Equation (1). *Calculation of Contrast-to-noise ratio (CNR).*

(1) $$\text{CNR}=\frac{\left|S_{A}-S_{B}\right|}{\sqrt{\sigma_{A}^{2}+\sigma_{B}^{2}}}$$

### 2.6 Immunohistochemical analysis

For immunohistochemistry, paraffin-embedded sections fixed in formalin 4% supplemented with 0.05% glutaraldehyde from human cornea tissue were analyzed by classical Haematoxylin & Eosin (H&E), and immunostaining for collagen I (Thermo Fisher Scientific, MA, USA). Shortly, primary antibody incubation was carried out at room temperature (r.t.) for 30 min, followed by a secondary incubation at r.t. for 30 min, and 10 min for chromogen substrate incubation with DAB Quanto (Thermo Fisher Scientific, MA, USA) at r.t., counterstaining with H-E for contrast and mounting with Pertex® (Histolab®, Askim, Sweden) was performed. A Zeiss fluorescent microscope was used to observe and document the outcomes.

### 2.7 SEM/EDXS analysis

For SEM/EDXS analysis, human cornea tissue fixed in formalin 4% supplemented with 0.05% glutaraldehyde that had undergone previous micro-CT analysis and that had subsequently been enriched with iodine and PTA contrast agents were examined with Hitachi Analytical TableTop Microscope/Benchtop SEM TM3030, in conjunction with EDS equipment (Bruker Nano GmbH, Berlin, Germany). Samples were paraffin-embedded after CT scanning, sectioned in 5-μm thick slices and attached on aluminum stubs using double-sided carbon tape. The contrast enhancement achieved through prior PTA treatment for CT scanning was found to be sufficient for the necessary conductivity during SEM/EDXS analysis. An acceleration voltage of 15 kV was used with a working distance of 9800 μm and magnification from 1,000× to 1,200× for imaging and EDXS mapping of carbon, iodine, and tungsten in the sample.

## 3 Results

### 3.1 Structural analysis of corneal tissue

**Fig. 1:**
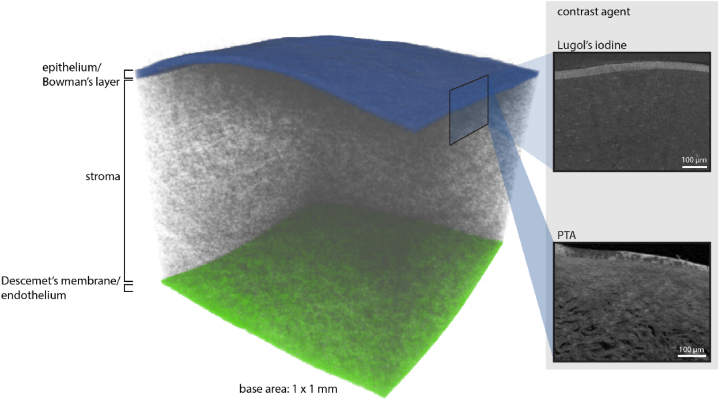
3D structure of the human cornea visualized by Nano-CT. Different contrast-enhancement agents for better visualization of the cornea structure.

Scans of corneal tissues unveiled the expected layers that can be identified as epithelium, including the Bowman's layer, stroma and the endothelial layer including the Descemet's membrane (Fig. 1). In contrast, treatment with the Lugol's iodine highlighted the epithelial and endothelial layer as well as stromal cells, the PTA increased the contrast of the CFs. The iodine-based contrast-enhancement agent was used to segment the epithelial (blue) and endothelial layer (green).

**Fig. 2:**
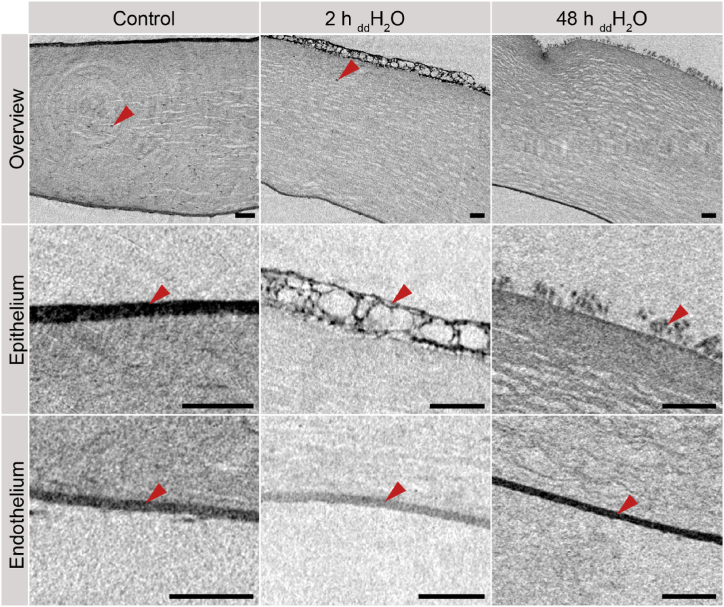
Visualization of the stroma and stromal cells (top row, arrow) corneal epithelium (middle row, arrow), and corneal endothelium (bottom row, arrow) using Nano-CT with iodine-based contrast enhancement. Scale bar: 100 μm.

Fig. 2 shows the corneal tissues contrast-enhanced using Lugol's iodine under the different swelling conditions in inverted colors. The number of visual stromal cells decreased with increased deionizing duration and completely disappeared after 48 h in deionized water. Whereas the structure of the endothelial layer was mainly retained, the epithelial layer became porous after 2 h incubation in deionized water and showed mostly detachments after 48 h. The porosity of the stroma increased visually with increased incubation time in deionized water. However, the contrast of the stromal tissue is comparatively low with the previous treatment (CNR = 0.72 ± 0.09, cf. Table 1). In higher magnification of the epithelial and endothelial layer (Fig. 2, row 2 and 3), the cell nuclei can be recognized. In the endothelial layer, the shape of the nuclei remained spherical, whereby the nuclei in the epithelial layer expressed an elongated structure after 2 h, especially in the connecting braces surrounding a pore. After 48 h the nuclei showed a spherical shape again, but showed a protruding appearance from the stromal tissue.

**Table 1:** CNR for different tissues and contrast enhancement protocols.

Contrast enhancement	Lugol's iodine 1.5%			PTA 0.3%		
Tissue	Stroma	Endothelium	Epithelium^a^	Stroma	Endothelium	Epithelium^a^
CNR ± STD	0.72 ± 0.09	3.64 ± 0.13	3.41	3.90 ± 0.50	6.01 ± 0.27	2.96

a CNR based on Control (n = 1).

Based on the 3D data sets represented by the 2D sections in Fig. 2, epithelial and endothelial thicknesses were determined. The stromal thickness was calculated as the spatial distance between the epithelial and endothelial layers. The stromal surface was used as a distance marker for the caused detachment of the epithelium after 48 h incubation in deionized water. Consequently, the epithelial thickness after 48 h was not determined.

**Fig. 3:**
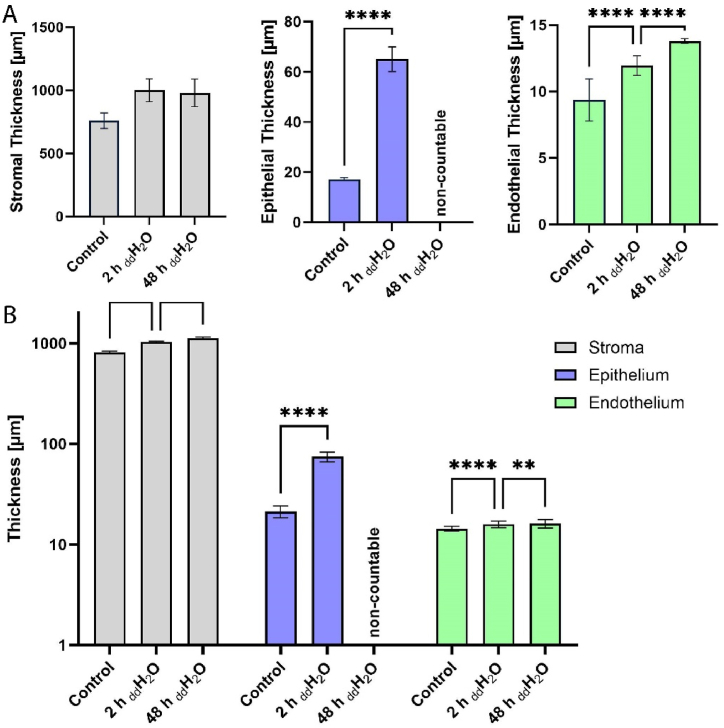
Quantitative analysis of the corneal thickness divided into epithelial, stromal and endothelial portions under different swelling conditions. **(A)** Thickness measurements derived from 3D segmentation (n > 1200). Values are mean ± SEM. Statistical analysis by Tukey test and 2-way ANOVA, *p < 0.0332, **p < 0.0021, ***p < 0.0002, ****p < 0.0001. Stromal thickness was measured as a spatial distance by ‘distance map’ without ANOVA. **(B)** Thickness measurements obtained from 2D cross-sections (n = 15). Values are mean ± SEM. Statistical analysis by Tukey test and 2-way ANOVA, *p < 0.0332, **p < 0.0021, ***p < 0.0002, ****p < 0.0001.

The quantification of the layer thicknesses shows that the epithelium increased in thickness by 48 μm in 3D measurements (280%) (Fig. 3A) and 54 μm in 2D measurements (Fig. 3B) after 2 h in deionized water. The stromal tissue swelled by only 26% and retained approximately the thickness after 48 h. Swelling of the endothelial layer could be detected as 28% after 2 h and 47% after 48 h based on the thickness of the control. However, the thickness homogeneity increased with a longer incubation duration indicated by a lower standard deviation in 3D measurements.

**Fig. 4:**
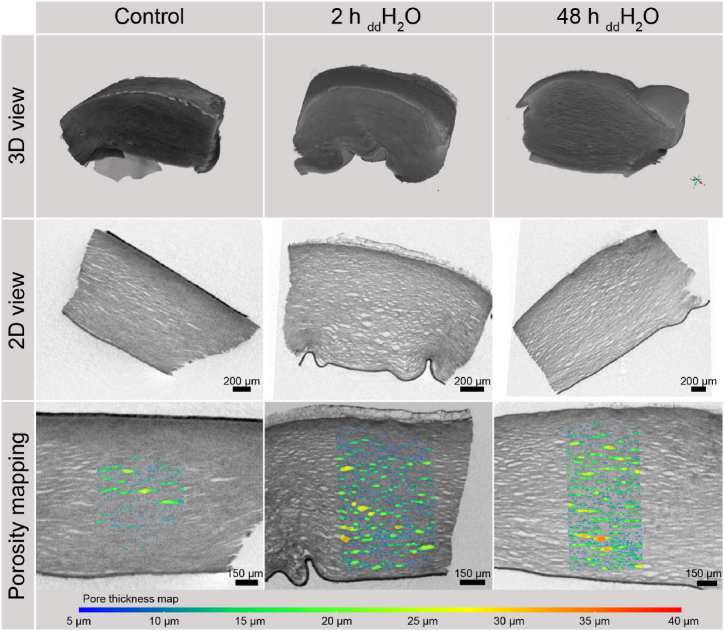
Visualization of the cornea using micro-CT with PTA-based contrast enhancement. The top of each sample indicates epithelial and bottom endothelial layer.

Additional PTA-based contrast enhancement of the specimen shown in Fig. 2 leads to high contrast of the corneal stroma (CNR = 3.90 ± 0.50, cf. Table 1). Fig. 4 illustrates 3D cross-views (top) and a 2D sections (middle) of the PTA contrast-enhanced cornea under the different swelling conditions. Intermediate spaces caused by the swelling and collagenous tissue could be segmented, whereas the endothelium and epithelium show similar attenuation to the stromal tissue. In particular, stromal tissue close to the epithelium shows higher attenuation than in the middle area of the stroma. Porosities of stromal tissue increased with longer incubation time in deionized water from 4.30% (Control) to 16.31% (2 h in deionized water) to 19.35% within 48 h in deionized water. The strong increase of porosity from control to 2 h in deionized water and the smaller increase from 2 h to 48 h incubation show consistent findings with the considerations of swelling of stromal tissue shown in Fig. 3. Fig. 4 (bottom) indicates the distribution of the pore spaces between the fibers in the stroma for a section in the sample using volume mapping of the segmented pores.

**Fig. 5:**
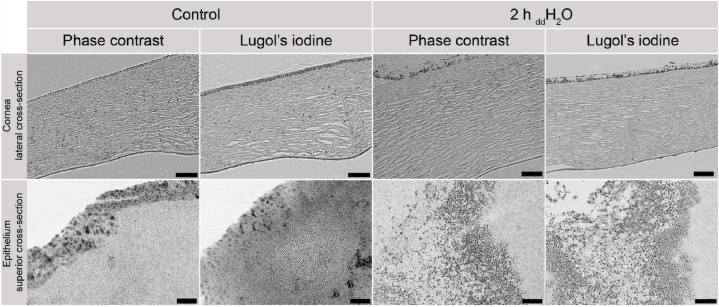
Visualization of the cornea using phase contrast imaging, both with and without Lugol's iodine contrast enhancement. The top row presents lateral cross-sections of the cornea, comparing non-hydrated (left) and 2-h hydrated specimens (right) using phase contrast alone (each left) and phase contrast combined with contrast enhancement (each right). The bottom row provides a superior cross-section view of the epithelium. Scale bar: 100 μm.

Synchrotron X-ray computed micro-tomography scans of the human cornea, both with and without the use of the contrast agent Lugol's iodine, are displayed in Fig. 5. The structural organization of the tissue remained qualitatively similar as found in the contrast-enhanced micro-CT scans presented in Fig. 2.

In both the control group and samples treated with deionized water for 2 h, stromal cells are visible, albeit with reduced prominence in the latter. Notably, stromal layer porosity increased with hydration. Furthermore, the epithelium signifiantly increased in thickness and porosity with hydration.

Examining the bottom row of Fig. 5, superior cross-sections indicated distinctive cell arrangements in the epithelium with and without hydration. Without induced hydration, epithelial cells were densely packed, while the epithelium subjected to 2 h of hydration displayed spherical interspaces between cells, primarily in the outer region. Conversely, the region adjacent to the stroma maintained its densely organized structure.

**Fig. A1:**
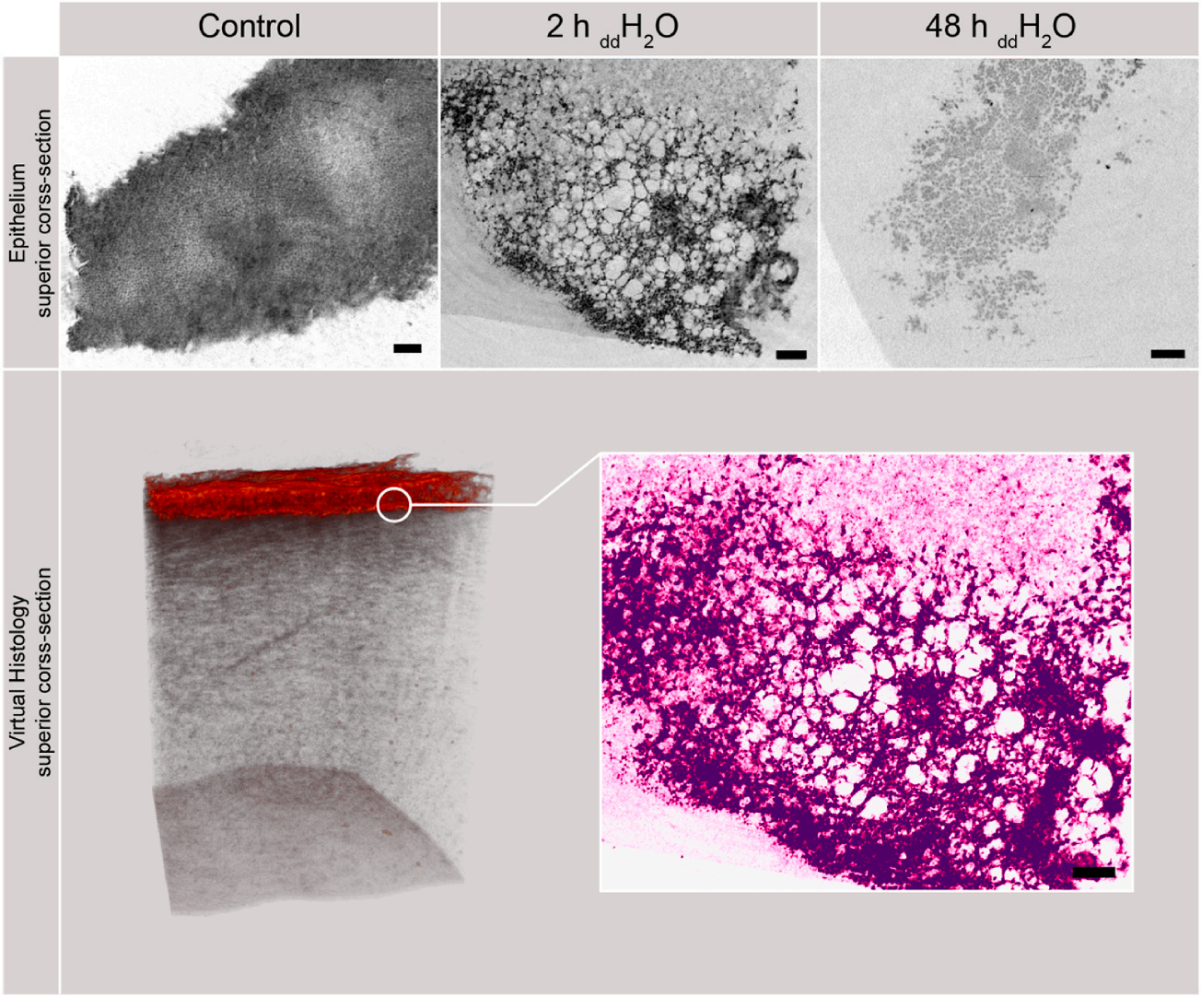
Visualizing structural changes in the corneal epithelium due to hydration. The top row displays superior cross-sections of the epithelium of non-hydrated (left), 2-h hydrated (middle), and 48-h hydrated specimens (right) using contrast-enhanced micro-CT with Lugol's iodine as contrast agent (inverted grey values). Virtual histology enables digital sectioning in any direction, facilitating superior cross-sectional views of thin layers. By applying colors, an appearance comparable to Haematoxylin and Eosin (H&E) staining can be achieved (bottom). Scale bars: 100 μm.

As observed in contrast-enhanced micro-CT scans (Figure A1), superior cross-sections through the epithelium yielded congruent results.

### 3.2 Evaluation of the contrast enhancement

The CNR of the corneal tissues were calculated according to Equation (1). Table 1 shows the obtained CNR for each tissue and contrast enhancement, whereas the three scans calculated each value and thus different swelling conditions. The CNR of the epithelium is only based on the projections of the control group due to increasing detachment after incubation in deionized water. Endo- and epithelium show high CNR in both contrast-enhancing protocols. Corneal stroma showed a low CNR with Lugol's iodine, but a high CNR after contrast enhancing with 0.3% PTA. In contradiction with a higher visual contrast of the epithelium, the CNR is lower than the endothelium's CNR. This is caused by higher corresponding noises of the signal $\sigma_{epithelium}$ in both groups, the Lugol's iodine and PTA treated.

### 3.3 Immunohistochemical analysis

**Fig. 6:**
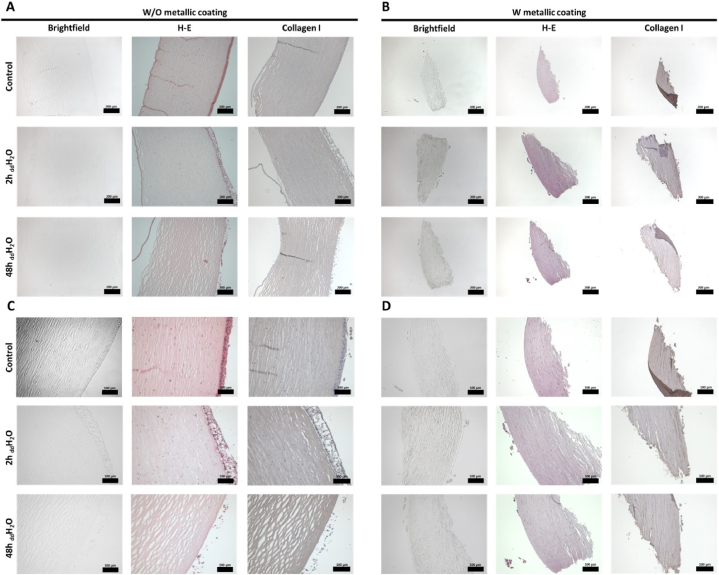
Comparison of the Nano-CT nanostructural features to the immunohistochemical (IHC) analysis of the corresponding corneal tissue under different swelling conditions (IHC for cornea epithelial, stroma, and endothelial cells, as well as collagen fibers). (**A, C**) Cornea sections, and (**B, D**) metalized cornea sections in brightfield, stained with haematoxylin and eosin, and collagen I at 10× (**A, B**) and 20× (**C, D**). The presence of the protein is marked by the brown dark color. The scale bars are 200 (**A, B**) and 100 (**C, D**) μm. (For interpretation of the references to color in this figure legend, the reader is referred to the Web version of this article.)

The human corneas pre- (Fig. 6A and C) and post-metallization (Fig. 6B and D) were stained by Haematoxylin and Eosin, and collagen I. The latter was lower in the samples with metallic coating compared non-metalized ones (Fig. 6). These corneal tissues showed an increase in thickness due to the _dd_H_2_O immersion effect, to eventually a completely lose its epithelium and endothelium (Fig. 6).

### 3.4 SEM/EDXS analysis

**Fig. 7:**
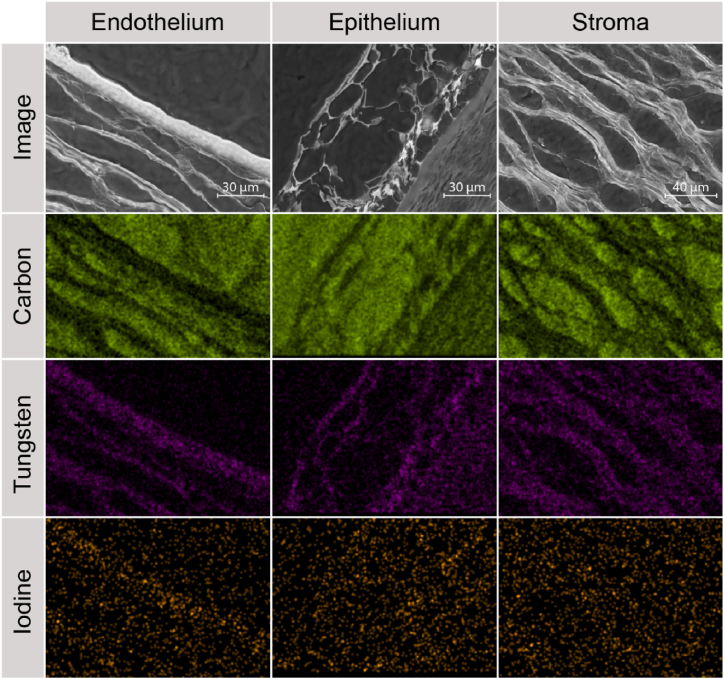
SEM/EDXS analysis of the corresponding corneal tissue with 2 h of hydration. Epi- and endothelium as well as stroma showed tungsten. Iodine was measurable in the endothelium and epithelium. Carbon indicated the paraffin embedding.

SEM imaging of the corneal tissue showed the three different layers of endothelium, epithelium, and stroma in samples treated with deionized water for 2 h (Fig. 7). Epithelium for samples with 48 h treatment in water was not evident (data not shown). EDX/S mapping of carbon showed the double-sided carbon tape and paraffin embedding as background, whereas tungsten is present in the entire sample, including endo- and epithelial as well as stromal tissue. The signal for iodine needed to be stronger. However, an increased signal is indicated in the endothelium and less evident epithelium, predominantly in the region facing the stroma.

## 4 Discussion

Contrast-enhanced micro-CT of the human cornea reveals the layers of the cornea, including the epithelium, stroma, and the endothelial layer allowing for a three-dimensional structural analysis at different degrees of hydration as shown in Fig. 1, Fig. 2, Fig. 3, Fig. 4. By employing pixel sizes of 800 nm, the presence of nuclei and thus cells in the stroma as well as epithelium could be identified, indicated with arrows in Fig. 2 (top row). Cells in the endothelium could not be distinguished, displayed as a homogenous structure due to limited resolution and the densely packed cells. Studies investigating nuclear contrast enhancement using lead(II)acetate, including Axolotl larva forelimb samples, allowed the differentiation of tissues according to the observed local cell density [27]. However, in regions of high cell densities, it is challenging to distinguish singular cells similar to the cells in the endothelium. Contrary to the densely packed endothelium (Fig. 2, bottom), the visibility of single cells in the hydrated epithelium and stromal layer increased.

Layer thicknesses of the corneal tissue and porosity of the stroma could also be determined using an epi- and endothelium segmentation to measure the thickness using volume thickness mapping of the segmented layers as illustrated in Fig. 3A. The stroma thickness in corneas was determined by measuring the distance between the segmented epi- and endothelium. This is based on a sphere-fitting method that reduces the user-induced bias of histological processing, as reported in approaches with light sheet microscopy for vascular injury [28]. The sphere-fitting approach results in accordance to measurements obtained from 2D measurements from aligned cross-sections (Fig. 3B). Conventional histology and measurements of thin layers, on the other hand, are affected by the compressive strains in the sectioning direction and in the direction perpendicular to sectioning [29] as well as the orientation in the embedding. A 3D analysis of layer thickness is, therefore, less susceptible to distortions due to its non-destructive nature [29]. However, the curled macrostructure of the cornea led to varying thicknesses within the sample position and thus increased standard deviations.

Contrast-enhancement agents such as Lugol's iodine and phosphotungstic acid (PTA) were applied successively to the human cornea to improve visibility in micro-CT imaging. Contrast-to-Noise Ratio (CNR) was calculated for each of the three corneal tissues epi- and endothelium and stroma to evaluate the effectiveness in visualizing the corneal structures. Lugol's iodine predominately enhanced the contrast of cellular tissues, such as epi- and endothelium, but not the collagen. Stromal cells in the non-treated and in the 2 h hydrated cornea were visualized with Lugol's iodine supporting the observation that the agent enhances the contrast of cells, but not of collagen. Reichardt et al. stated that iodine seems to lack the specificity to bind to collagen, resulting in blurry reconstructions and random clusters at a few tissue regions [30]. However, our scans did not reveal randomly enhanced clusters in collagenous regions, but showed low binding affinity to collagen.

PTA increased the contrast of the stroma to the same extend as Lugol's iodine for the epi- and endothelium in the applied manner. However, the contrast of the endothelium was increased further by applying the second contrast agent. The binding affinity of PTA to collagenous tissue is broadly reported in the literature [[31], [32], [33], [34], [35]]. Moreover, the amount of bound PTA measured by micro-CT was found to correlate with the collagen content measured from conventional histology [36]. Although the scans for the structural analysis of the collagen were made in pixel sizes of 1.62 μm and thus led to poorer resolutions compared to previous scans treated with Lugol's iodine, one can notice that the visibility of cells within stromal tissue would have been diminished yet at better resolutions. However, PTA as a contrast agent was found to be suitable for stromal analysis and provided higher contrast of the collagenous network than contrast enhancement with Lugol's iodine as shown in Table 1 and allows insights into the fiber orientation.

Synchrotron radiation can be utilized to image biological samples with poor absorption contrast using the phase-contrast technique. Human cornea without prior contrast enhancement could be imaged using the phase contrast. However, contrast enhancement with Lugol's iodine further improved the contrast of cellular structures in synchrotron X‐ray computed micro‐tomography. The applied voxel sizes in the synchrotron X‐ray computed micro‐tomography (Fig. 5) and the contrast-enhanced micro-computed tomography (Fig. 2) amounted to 0.9 and 0.8 μm, respectively, leading to a comparable degree of detail. However, edges were better defined and background noise was reduced in images using synchrotron radiation. Enhanced edge clarity could be particularly observed in the visualization of the non-hydrated epithelium (superior cross-section) containing densely arranged cells (Fig. 5 and Figure A1). This can be explained by the phase-contrast effects, but also by the preparation technique which included paraffin embedding to stabilize the sample, reducing the surrounding media's attenuation and enhancing phase contrast simultaneously. Due to the dehydration of the samples for embedding in paraffin, tissue shrinkage can be expected. In histological evaluations, a shrinkage of more than 14% could be measured [37], other studies using paraffin embedding for micro-CT soft tissue analysis resulted in 19.2–61.5% decreased volume [38]. Schned et al. showed that shrinkage on microscopic slides after rehydration was similar to the shrinkage caused by the fixation procedure [37]. However, scanning in paraffin will show the mentioned shrinkage above and thus distorted structural findings without using previously determined correction factors.

The corneal stroma naturally has a propensity to absorb liquid and enlarge, which results in it losing transparency [12,39,40]. Our data indicate a rise in corneal thickness during swelling, with a particular emphasis on the epi- and endothelium. These findings are consistent with prior studies in which the tissue was immersed in distilled water for one to four days [41]. The use of double-deionized water increases corneal thickness by 30% without appearing harmful to the endothelium. However, many scientists have noted that when the cornea swells, water may penetrate different compartments, including those between lamellae, into devoid of fibrils (often referred to as ‘Lakes’), between the fibrils within the lamellae, or within the fibrils themselves [18,19,42,43].

EDXS measurements revealed tungsten and iodine distribution in the contrast-enhanced corneal tissue, with tungsten detected throughout and iodine mainly in high cell-density areas. Carbon mapping served as a control for background and paraffin embedding. The distribution aligns with micro-CT images, though iodine signals were weaker, possibly due to lesser tissue attachment or diffusion out of the sample during storage in deionized water. This effect was likely exacerbated by water's role as a leaching agent, increasing contrast agent diffusion and displacing iodine due to osmotic imbalances between the sample and medium [44]. Notably, Lugol's iodine showed reduced durability in hydrogel embedments like agarose [45].

Our study utilized contrast-enhanced micro-CT to elucidate the ultrastructural alterations in human corneas—namely, the epithelium, stroma, and endothelium—under swelling conditions. This investigation significantly augments the existing body of research in ocular biology and biomechanics. Prior studies have already probed into the swelling-induced modifications in corneal structures across multiple mammalian species, illuminating changes in collagen fibril arrangements and cellular distribution [5,8,10,46]. They emphasized the intricate relationship between water content, collagen fibril arrangement, and corneal transparency. Earlier research has posited that water can infiltrate the collagen fibrils during swelling [39].

Furthermore, the critical role of the endothelial pump in regulating corneal hydration and maintaining transparency has been well-documented [41]. Our research extends these findings by employing state-of-the-art micro-CT imaging. In other attempts investigating the corneal ultrastructure, Epah et al. utilized Isolectin GS-IB4 A647 with a fluorescent label for *in vivo* vascular mapping via 3D light sheet ultramicroscopy (UM) and micro-CT. At the same time, post-mortem CT scans employed a radiopaque contrast medium. UM imaging facilitated the 3D visualization of vascular networks in subjects injected with the labeled Isolectin GS-IB4. This method was sensitive enough to assess individual endothelial cell involvement in capillary structures within Matrigel plug assays. However, more extensive vessel imaging was less effective due to selective endothelial staining.

Conversely, micro-CT and nano-CT were ineffective for Epah et al. for capillary imaging because of inadequate X-ray absorption and poor signal-to-noise ratios [47]. Morishige et al. investigated 27 healthy human corneas that underwent Descemet's stripping automated endothelial keratoplasty (DSAEK). Using second harmonic generation (SHG) microscopy, 3-mm square blocks from the central cornea were meticulously analyzed. The study aimed to measure the angles of collagen lamellae, both near the corneal surface (at 30 μm depth, known as sutural lamellae) and at deeper levels (50 or 100 μm) [48]. SAXS investigations have assessed collagen fibril arrangement within the human corneal stroma under various pathological conditions, frequently linked to deficiencies in proteoglycans [49].

Furthermore, this methodology has enabled the characterization of collagen's ultrastructural aspects, encompassing fibril spacing and diameter, across the human cornea. This analysis was conducted with a spatial resolution of 0.4–1.0 mm, comparing central and peripheral corneal tissue, as elucidated by Boote et al. [50]. The results unveiled a complex, 3D interweaving pattern of collagen lamellae within the anterior stroma of normal human corneas. Notably, these collagen structures exhibited strong adherence to Bowman's layer. This structural arrangement likely plays a crucial role in imparting rigidity and shaping the curvature of the cornea's anterior segment [51].

Our study showed significant swelling-induced changes in all corneal layers, with the epithelium thickness increasing notably after 2 h in deionized water and more moderate changes in the stroma and endothelium. We used immunohistochemical assays and scanning electron microscopy with energy-dispersive X-ray spectroscopy (SEM/EDXS) alongside micro-CT to link structural changes with molecular markers and elemental composition, particularly noting variations in collagen I during corneal swelling. This research offers vital insights into the ultrastructural changes in corneal tissues under swelling and hydration, highlighting the need to further explore corneal biomechanics and tissue behavior [41].

Future research should focus on translating lab results to clinical settings by developing predictive models considering patient-specific conditions, including postoperative scenarios, to support personalized medicine. This study provides a foundation for future research to improve clinical relevance and understanding of corneal biomechanics, aiding in developing better diagnostic and therapeutic strategies for ocular diseases. 3D reconstruction of corneal ultrastructure is valuable for diagnosing and predicting both normal and pathological tissues.

Future studies could explore dynamic tissue alterations beyond swelling to replicate physiological and pathological conditions more accurately. Experiments could include modulated swelling rates to mimic natural hydration variations, such as during sleep or environmental changes. Additionally, understanding the impact of aging and pathological conditions is crucial, as corneal collagen and proteoglycans changes due to aging or diseases like keratoconus and corneal edema significantly affect corneal biomechanics. These specific effects warrant thorough investigation [52,53].

The present study also opened the possibilities for evaluating the impact of pharmaceutical or therapeutic interventions on corneal swelling [54,55]. Subsequent research should focus on assessing the effectiveness of various treatment modalities, such as cross-linking for keratoconus or pharmacotherapy for corneal edema [[56], [57], [58]]. We also foresee further advances in corneal disease diagnostics, where one can identify correlations between specific structural shifts observed via micro-CT imaging and clinical manifestations of corneal diseases. Such intervention could lead to earlier detection and targeted monitoring of corneal pathologies [59]. Also, integrating complementary imaging techniques, such as advanced microscopy or spectroscopy, alongside contrast-enhanced micro-CT could offer a panoramic perspective of corneal tissue behavior [60]. A multi-modal approach will likely yield a more profound understanding of underlying biochemical processes.

Our study presents both advantages and limitations. Scanning biological tissues in an aqueous environment prevents dehydration-induced shrinkage [31,32] but is unsuitable for synchrotron beams due to bubble formation, which can create artifacts and damage samples [61]. Paraffin embedding avoids this issue but may alter the original ultrastructure due to required dehydration. Phase contrast micro-CT enhances cell visibility, and its combination with contrast agents further improves image quality. However, using desktop micro-CT systems like Skyscan 2211 in water increases background noise. Similar scanning methods to synchrotron radiation could enhance contrast but risk compromising ultrastructure [38]. Additionally, the resolution limits in absorption and phase-contrast micro-CT make distinguishing densely packed cellular structures challenging.

## 5 Conclusion

This study presents a comprehensive compilation of research in the field of corneal biology and imaging. It highlights the significance of advanced imaging techniques in unraveling the intricacies of corneal structure. It provides valuable insights for researchers and clinicians working in the field of ophthalmology and biomedical engineering. This research leverages the innovative approach of contrast-enhanced micro-CT to provide unprecedented insights into the three-dimensional distribution of cells within the hydrated cornea. The implications of this work are of significant importance for advancing our understanding of corneal biology and its potential applications in clinical settings.

In conclusion, this study not only showcases the potential of contrast-enhanced micro-CT as a powerful tool for visualizing and analyzing biological tissues but also highlights its specific relevance studying the human cornea. The findings presented in this study have the potential to influence further research directions in ophthalmology and related fields. Furthermore, they open up possibilities for improving our understanding of corneal structure and function, which may ultimately contribute to advancements in diagnosing and treating corneal disorders. This research stands as a noteworthy contribution to biomedical imaging, underscoring the importance of innovative imaging techniques in advancing our knowledge of complex biological structures, making it a valuable resource for the optometry and vision science community.

## Ethics declaration

The Regional Committee for Medical and Health Research Ethics in South-Eastern Norway (No 2017/418) approved tissue harvesting and laboratory procedures, and all tissue collections complied with the Guidelines of the Helsinki Declaration.

## CRediT authorship contribution statement

**Gerard Boix-Lemonche:** Writing – original draft, Visualization, Investigation. **Torben Hildebrand:** Writing – original draft, Visualization, Investigation, Formal analysis. **Håvard Jostein Haugen:** Writing – review & editing, Resources, Conceptualization. **Goran Petrovski:** Writing – review & editing, Resources, Conceptualization. **Liebert Parreiras Nogueira:** Writing – review & editing, Supervision.

## Declaration of competing interest

The authors declare that they have no known competing financial interests or personal relationships that could have appeared to influence the work reported in this paper.
